# Unmasking the Twitter Discourses on Masks During the COVID-19 Pandemic: User Cluster–Based BERT Topic Modeling Approach

**DOI:** 10.2196/41198

**Published:** 2022-12-09

**Authors:** Weiai Wayne Xu, Jean Marie Tshimula, Ève Dubé, Janice E Graham, Devon Greyson, Noni E MacDonald, Samantha B Meyer

**Affiliations:** 1 Department of Communication University of Massachusetts Amherst Amherst, MA United States; 2 Department of Computer Science Université de Sherbrooke Sherbrooke, QC Canada; 3 Axe maladies infectieuses et immunitaires, Centre de Recherche du CHU de Québec Laval University Quebec City, QC Canada; 4 Direction des risques biologiques et de la santé au travail Institut National de Santé Publique du Québec Quebec, QC Canada; 5 Department of Pediatrics Dalhousie University Halifax, NS Canada; 6 School of Population and Public Health University of British Columbia Vancouver, BC Canada; 7 School of Public Health Sciences University of Waterloo Waterloo, ON Canada

**Keywords:** infoveillance, data analytics, Twitter, social media, user classification, COVID-19

## Abstract

**Background:**

The COVID-19 pandemic has spotlighted the politicization of public health issues. A public health monitoring tool must be equipped to reveal a public health measure’s political context and guide better interventions. In its current form, infoveillance tends to neglect identity and interest-based users, hence being limited in exposing how public health discourse varies by different political groups. Adopting an algorithmic tool to classify users and their short social media texts might remedy that limitation.

**Objective:**

We aimed to implement a new computational framework to investigate discourses and temporal changes in topics unique to different user clusters. The framework was developed to contextualize how web-based public health discourse varies by identity and interest-based user clusters. We used masks and mask wearing during the early stage of the COVID-19 pandemic in the English-speaking world as a case study to illustrate the application of the framework.

**Methods:**

We first clustered Twitter users based on their identities and interests as expressed through Twitter bio pages. Exploratory text network analysis reveals salient political, social, and professional identities of various user clusters. It then uses BERT Topic modeling to identify topics by the user clusters. It reveals how web-based discourse has shifted over time and varied by 4 user clusters: conservative, progressive, general public, and public health professionals.

**Results:**

This study demonstrated the importance of a priori user classification and longitudinal topical trends in understanding the political context of web-based public health discourse. The framework reveals that the political groups and the general public focused on the science of mask wearing and the partisan politics of mask policies. A populist discourse that pits citizens against elites and institutions was identified in some tweets. Politicians (such as Donald Trump) and geopolitical tensions with China were found to drive the discourse. It also shows limited participation of public health professionals compared with other users.

**Conclusions:**

We conclude by discussing the importance of a priori user classification in analyzing web-based discourse and illustrating the fit of BERT Topic modeling in identifying contextualized topics in short social media texts.

## Introduction

### Background

The COVID-19 pandemic is a crisis that has taken millions of lives, devastated the world economy, and disrupted almost every aspect of human society. Mask wearing is one of the few early and effective nonpharmaceutical interventions to curb the spread of the virus [1,2]. However, public health efforts to mandate or recommend mask wearing have been met with public skepticism [3], and in some cases, outright resistance. This could be a result of mixed messaging—early in the pandemic, some public health institutions (eg, World Health Organization and US Centers for Disease Control and Prevention) and media advised against mask wearing, citing concerns regarding mask shortage for health care workers and the efficacy of masks [4]. It could also have resulted from widespread unproven medical claims from many conservative media outlets and influencers [5]. The effectiveness of mask wearing to prevent transmission of SARS-CoV-2 has been much debated as the scientific literature has evolved rapidly, and messages from official government and medical advisory bodies have been mixed since the early days of this pandemic; it is also likely that fierce antimask sentiment more closely reflects deeply rooted anti-Asian racism and xenophobia [6], as well as populist and nativist resentments [7]. Populist leaders and parties sought to politicize mask wearing calling the public health response to the pandemic government overreach and a conspiracy [8]. Armed protests against mask wearing were held across US cities. National surveys demonstrate a clear link between political-right partisanship and Christian-nationalist ideologies and resistance to government-mandated COVID-19 restrictions [9]. Understanding the political context within which public health measures and messaging are being implemented is critical to maximizing the success of attempts to protect population health. Infoveillance based on web-based discourse provides ways to understand the political nature and implications of public health issues. Although there is a growing body of infoveillance studies that leverage the latest digital analytic tools to document and compare public health discourse, we notice several gaps. This project seeks to present an improved infoveillance framework to understand public health discourse varied by political and apolitical groups. This paper was organized as follows. We first situate the case study of mask wearing in the context of medical populism, followed by the introduction of infoveillance. We then proceed to 2 existing gaps in the existent literature, leading to our proposed computational framework.

### Medical Populism

There are growing calls to study the politicization of public health issues to understand competing interests and ideologies in public health measures. The COVID-19 pandemic presents an interest case of *medical populism* [7,10,11], defined as “as a political style based on performances of public health crises that pit ‘the people’ against the dangerous others, which consists of ‘the establishment’” [11]. A common thread in populism is the dichotomy between virtuous people and the elite or establishment, which is perceived as corrupt [12]. In the medical populism regarding Ebola, HIV, drug addiction [11], and the antivaccination movement [13], the medical and scientific communities are framed as elites to be blamed and distrusted. Recent surveys show that populist ideology is associated with a higher degree of distrust in political and scientific institutions, leading to a heightened acceptance of COVID-19–related conspiracy theories [14], with such distrust associated with a lower level of education, health literacy, and the use of logic thinking [15,16]. Such distrust of elites and institutions is fertile ground for those peddling alternative and unproven medicines such as hydroxychloroquine, which have been endorsed by populist leaders including Donald Trump and Jair Bolsonaro [17]. Medical populism breeds disinformation and misinformation, which is made worse by viral transmission on social media [18]. Although not unique to the COVID-19 pandemic—misinformation was rampant during the flu pandemic [19], as well as Zika [20] and Ebola [18] outbreaks—the level of politicization and social media involvement led the World Health Organization to establish a task force on the infodemic [21], and some experts call the COVID-19 pandemic the first true social media infodemic [22].

### Infoveillance and the COVID-19 Pandemic

Social media provides the public with fodder for civic deliberations and actions. A wealth of research theorizes social media’s role as a mediated public sphere or the nexus of networked societies [23]. This trove of social media data, indicative of public attention, attitudes, and actions, can be readily tapped into for infoveillance. Infoveillance is a methodological framework that uses large-scale digital behavioral data to monitor outbreaks and public perceptions of public health issues [24-26]. There are successful implementations of infoveillance in past epidemic outbreaks, including Ebola [27], Zika [28,29], and H1N1 influenza [30].

Our review of the growing body of infoveillance research since the COVID-19 outbreak revealed 3 common themes. First, studies using data from the early stage of the COVID-19 outbreak aim to detect linguistic and content features in social media texts that are predictive of COVID-19 symptoms [31,32]. This approach is in line with traditional infoveillance projects, such as the pioneer, albeit flawed, Google Flu Trends, which became a famous example of “big data hubris” after initially appearing to predict influenza prevalence faster than traditional public health surveillance methods [33].

Second, as public conversations broadened, later studies used latent Dirichlet allocation (LDA) topic modeling to reach beyond mere mention-counting to identifying themes in web-based discourse. Chandrasekaran et al [24] identified COVID-19–related economic impacts, virus spreads, treatment and recovery, impact on the health care sector, and government’s response. Abd-Alrazaq et al [34] identified themes surrounding the origin of the virus; the impacts of COVID-19 on people, countries, and the economy; and mitigation and prevention. Similarly, Wahbeh et al [35] identified topics in digital texts that revolve around actions and recommendations, misinformation, knowledge, the health care system, symptoms and illness, immunity, testing, and infection and transmission. Although most studies relied on Twitter data, a few used Weibo, the Chinese microblogging site [32,36-38]. Weibo data revealed uncertainty and changing attitudes about the COVID-19 pandemic by the Chinese public in the early days of the outbreak [36]. Prior works also examined user sentiments [24,35,39]. Zhou et al [40] exemplify this, using Weibo data to monitor Chinese public response to lockdowns and how negative sentiments such as panic evolved.

Third, most prior works examine general discourses. Al-Ramahi et al [3] identify major themes in the antimask discourse, including constitutional rights and freedom of choice; COVID-19–related conspiracy theory, population control, and big pharma; and fake news, fake numbers, and fake pandemic. Relatedly, Doogan et al [41] tracked public responses to mask wearing and social distancing across 6 countries, finding that attention paid to public health measures correlated with case numbers. These studies, along with computational text analysis of news content from traditional media [42], contribute to the growing understanding of the interplay between public health and public opinion.

### Gaps in Infoveillance Studies: Ideology and Identity Politics

Audience segmentation is a popular method of understanding the complexity and diversity of the user ecosystem in web-based discourse. In general information science studies, data-driven personas play a vital role in predicting and aggregating user behaviors [43]. The data-driven personas approach includes using various social media data streams and interaction patterns to cluster users based on demographic factors and interests [44]. The approach also applies to the public health domain, such as using survey data to generate psychological and demographic profiles of the public in adopting COVID-19 recommendations [45]. In the infoveillance literature, there has been some research on discourse by different users across geographic locations and with different health profiles [46,47] as well as in various health care sectors [35]. However, these are limited attention to politically and socially defined user clusters including those associated with medical populism. A few exceptions include the study by Walter et al [48] of the Twitter discourse on vaccines. The authors used unsupervised machine learning and network analysis to identify politically different “thematic personas” and subsequently analyzed content by each thematic persona. This study took a similar approach, albeit with new analytic tools, to explore the political nature of public health discourse and users who participate in the discourse. This entails moving beyond the general discourse and focusing on specific user groups that differ by politics and interests.

Internet users, such as offline publics, commonly seek support and influence by forming close-knit and like-minded communities. We borrowed the term *issue publics* from the general social science literature to refer to web-based user clusters connected through common backgrounds, hobbies, interests, and ideologies [23]. Users connect not only through social media following and follower linkages but also, more broadly, through symbolic connective actions such as hashtags [49]. For instance, users who identified with a social cause or political party use shared hashtags (eg, #ChinaVirus or #KungFlu) as a form of expression, resistance, and solidarity building. Hashtags connect ideologically similar causes and weave disconnected local concerns and identities into a global narrative [49-51].

Previous studies view these politically connected user groups as *ad hoc issue publics* [23], *networked counterpublics* [50,51], or *countercoalitions* [52]. These user groups form quickly in response to developing news, emergent social movements, or long-held belief and social identities. They are decentralized, geographically distributed, and marked by coordinated sharing and discussions [23]. They consist of different institutional and individual stakeholders across public spheres, characterized by various levels of internal coordination and committed participation [52]. Terminology aside, the assumption is straightforward: the digital space is a web-based public square consisting of different user groups who have competing interests and ideologies. To understand how publics perceive public health measures, one must extract and triangulate discourse from each specific user group (ie, issue public).

### Gaps in the Current Infoveillance Studies: From LDA to BERT Topic Modeling

Current infoveillance studies overwhelmingly use LDA and sentiment scoring [53-54]. The reliance on LDA is not surprising, given it is the most popular and widely used topic model [55-58]. LDA is a probabilistic model that discovers latent topics in a text corpus and can be trained using collapsed Gibbs sampling [55,59,60]. Specifically, LDA assumes K underlying topics, each of which is a distribution over a fixed vocabulary. Although LDA is reputed to yield promising results in modeling text corpora [61], it fundamentally suffers from several shortcomings, including difficulty in setting the parameter *k*, which refers to the number of topics to yield semantically meaningful results, a deficiency in handling short texts [58], in capturing the contextual meaning of sentences [58], as well as its inability to model topic correlations and the evolution of topics over time [62].

To overcome these limitations, the new generation of topic models [56,57,61] use pretrained representations such as BERT to enable topic modeling (1) to consider contextual meaning of sentences for supporting the results to match the adequate topics and (2) to include more features for efficiently modeling topic correlations and topic evolution over time. Recent pretrained contextualized representations such as BERT have pushed the state of the art in several areas of natural language processing due to their ability to expressively represent complex semantic relationships from being trained on massive data sets. BERT is a bidirectional transformer-based pretrained contextual representation using masked language modeling objective and next sentence prediction tasks [62]. The significant advantage of BERT is that it simultaneously gains the context of words from both left and right context in all layers. To this end, BERT uses a multilayer bidirectional transformer encoder, where each layer contains multiple attention heads.

It is important to note that BERT is one of the latest unsupervised topic modeling techniques that seek to improve upon the traditional LDA approach. An alternative technique, the Analysis of Topic Model Networks (ANTMN), applies community-detection algorithms in network analysis to cluster LDA-generated topics [63]. ANTMN is a fitting tool for revealing framing in web-based and news discourse and has been used in studying public health discourse [64]. Another alternative is the semantic network–based classification algorithm textnets [65], which first uses LDA to cluster corpus into topics and then applies community-detection algorithms to categorize topics into network clusters. Although ANTMN and textnets are much-improved tools compared with the traditional LDA, we opted for BERT because BERT can reveal longitudinal topic trends, which is a feature not available in textnets, making BERT ideal for studying the ebbs and flows of specific topics in web-based discourse over time.

### Research Questions

This paper used topic modeling with BERT to overcome the incompatibility between traditional LDA methods and short texts (eg, tweets) and track topical evolutions longitudinally. In addition, we investigated discourses and topics unique to different user groups (ie, issue publics). This approach aimed to understand the role of political ideologies and political groups in defining the public health discourse.

Research question 1: How did English language Twitter discourse on masks and mask wearing change over the course of 2020?

Research question 2: How did English language Twitter discourse on masks and mask wearing vary across issue publics?

## Methods

With the focus on distinct user groups (ie, issue publics) and the state-of-art BERT Topic modeling application, this paper sought to present an infoveillance workflow consisting of data collection, data cleaning, and user classification and topic modeling.

### Data Collection

This study uses a large-scale COVID-19 Twitter corpus provided by Georgia State University’s Panacea Lab [66]. The corpus contains publicly available tweets from the Twitter Stream application programming interface (API) with the following keywords: “COVD19,” “CoronavirusPandemic,” “COVID-19,” “2019nCoV,” “CoronaOutbreak,” “coronavirus,” and “WuhanVirus.” We used a modified Python script to hydrate all COVID-19–related tweets sent between January 1 and December 31, 2020, based on the tweet IDs provided in the public data set.

### Data Cleaning

To track longitudinal trends associated with changes in developments related to the COVID-19 pandemic, our research team divided the COVID-19 data chronologically. Stage 1 spans the period from January 1 to April 30, 2020, including the early outbreak in China, subsequent travel restrictions and lockdowns first by China and later western democracies, and the emergent shortage of personal protective equipment (PPE). The cutoff date of stage 1 corresponds to when some major US states, such as Texas and Florida, started to relax public health measures in an effort to reopen the economy. Stage 2 spans May 1 to August 31, 2020, including events such as recommended or mandated mask wearing, armed protests against public health measures in the United States, controversial remarks about the COVID-19 pandemic by politicians, and the worsening pandemic in the English-speaking world. Stage 3 spans September 1 to December 31, 2020, during which significant political events in the United States include President Trump’s October contraction of COVID-19, the November presidential election, and the national vaccination campaigns. To identify the mask-related discourse, the following keyword filters were used: *mask*, *face cover*, *facecover*. To achieve computational efficiency (running BERT Topic models on a large corpus is time-consuming), we only kept English language tweets that received at least 1 retweet by other users to focus on tweets that are actually promoted to a wider audience. We also excluded tweets sent by users with blank Twitter user bio pages (to be explained in the *User Classification* section).

### User Classification

To identify the user classifications, we applied the k-means clustering algorithm [67] to Twitter users’ bio descriptions to classify users based on expressed identities and interests. With the focus on clusters of users who have expressed common interests and identities, users who had blank Twitter bios (0.54% of the total users) were excluded from the analysis. Although this exclusion may affect the representativeness of the discourse under study, we argue that users who use a common set of hashtags and terms in Twitter bios are more engaged (topically, socially, or politically) in this digital public square.

The k-means clustering algorithm was applied to yield 10 clusters. The algorithm put users who used similar words or phrases in Twitter bios in the same group, with the number of clusters (ie, 10) and the size of each cluster determined by the k-means algorithm. Researchers manually inspected the clustering output and removed 2 clusters mostly associated with news media and official sources (eg, Centers for Disease Control and Prevention, city and county governments) due to this study’s focus on citizens’ discourse. The remaining 8 clusters were reduced to 4 clusters based on topical similarity and political affiliation. The *general* cluster includes users whose Twitter bio descriptions indicate various social, professional interests, affiliations, and identities without sign of political affiliation. The *conservative* cluster includes users who use keywords and hashtags that indicate their conservative ideologies and support of the Trump administration (eg, #maga, #kag, #2a, or #prolife). The *progressive* cluster includes users who use hashtags and keywords reflecting a progressive ideology (eg, LGBTQI, Democrat, #BidenHarris2020, #Biden2020, or #BlackLivesMatter). Finally, the public health cluster includes users affiliated with the health care sector and public health research, as indicated by keywords such as *healthcare*, *science*, *epidemiologist*, *professor*, and *radiologist*. To provide descriptive findings on characteristics of each user cluster, we used a short text–classification algorithm called *textnets* to produce network visualizations of various phrases and hashtags used in Twitter bios [65]. This algorithm applies network analysis to natural language processing, providing an alternative to topic modeling for analyzing short texts such as Twitter bios. This approach can show latent identities, interests, and movements with which users in a particular cluster identify.

### BERT Topic Modeling

We removed all English stop words in the data set, using the natural language toolkit. We noticed a ubiquitous presence of the words “mask,” “covering,” “cover,” “face cover,” and “face mask” in the learned topics because our data set contained mask-related discourse. Practically, these words were noisy and degraded the performance of proper topics and hindered the interpretability of results. To overcome this problem, we extended the natural language toolkit vocabulary by adding these words and removed them in our data set. To identify potential topics within the mask-related discourse, we applied the BERTopic, a BERT-based topic modeling Python library. BERTopic extracts document embeddings using a pretrained BERT model. We used the BERT topic model, which comprises 12 layers, 12 attention heads, and 110 million parameters, to enable BERTopic to produce document embeddings to detect semantic similarity between sentences. BERTopic leverages BERT embeddings and a class-based term frequency–inverse document frequency to create dense clusters to detect unique topics. In addition, BERTopic generates the topic representations at each timestamp for each topic. The traditional LDA topic modeling requires a predefined k (the number of topics) for algorithms to cluster corpus around k topics [68]. BERTopic does not require a predefined k, reducing the need for various iterations of model fine-tuning.

### Ethics Approval

As we are using a publicly archived data set and no personally identifiable information is included nor published, we deem this research outside the purview of the institutional review board. Nevertheless, we have taken extra caution when analyzing each cluster’s user profiles to ensure that the reported data are aggregated and anonymous.

## Results

### Overview

**Figure 1:**
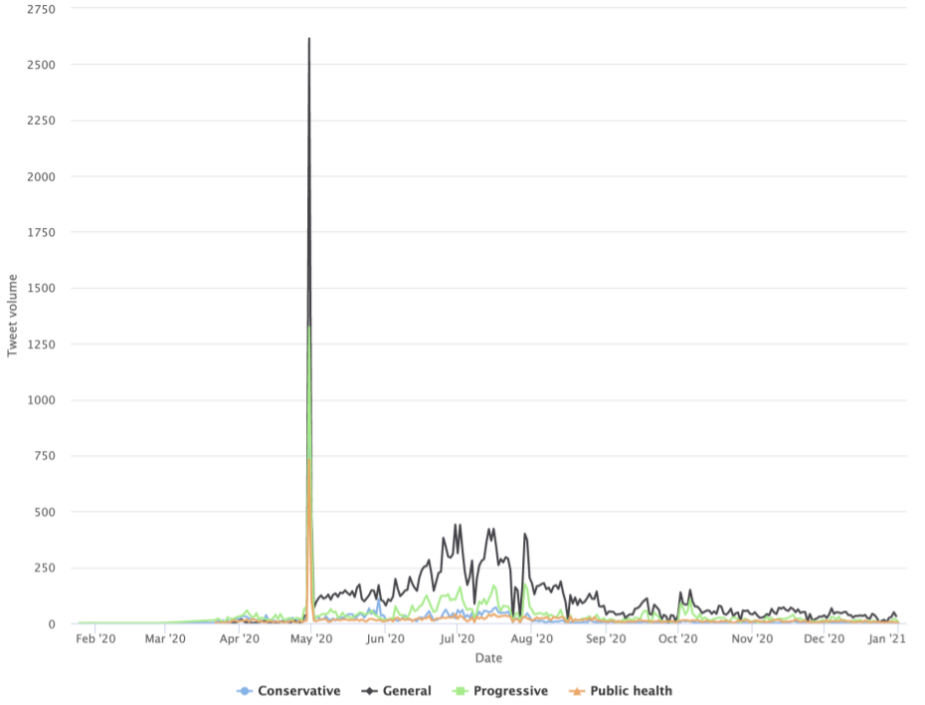
Volumes of topically classified tweets over time.

With the mask-related keywords applied as filters, the raw data set includes 1,061,686 unique tweets by 648,528 unique users in stage 1, includes 1,060,987 tweets by 576,274 unique users in stage 2, and includes 678,474 unique tweets by 359,561 unique users. Among them, stage 1 had 171,271 English language unique tweets that were retweeted at least once by 115,349 users; stage 2 had 234,997 unique English tweets by 137,426 users, and stage 3 produced 129,089 tweets by 76,443 users. As noted earlier, we also excluded tweets sent by users with blank Twitter bio pages. The final tweet data set before the user-classification scheme and BERT Topic modeling were applied included 163,378 tweets by 109, 097 users in stage 1, included 224,830 tweets by 129,830 users in stage 2, and included 123,843 tweets by 72,495 users in stage 3. This result focuses on tweets sent by the 4 identified user clusters: general, progressive, conservative, and public health. Figure 1 shows the tweet volumes by each distinct user community over time. There is a marked peak in tweet volume on April 30, 2020, across user clusters. The time corresponds to prominent US politicians’ mask-wearing practices, such as then Vice President Mike Pence’s mask wearing on April 30 when visiting a factory and his widely criticized maskless visit to Mayo Clinic on April 28.

To carry out topical analysis by user clusters, we ran BERT Topic models for each previously identified user cluster. Note that in the topic models some tweets were found to have no coherent theme and thus assigned to the unclassified topic −1 (the nonthematic). Following the common practice suggested by the authors of BERTopic, we did not include such nonthematic tweets in the final analysis. Table 1 shows the number of nonthematic tweets in each user cluster and the calculated ratios of nonthematic tweets. The nonthematic ratios vary across user clusters and stages. This shows the potential limitations of this topic modeling approach in that it leaves out some percentages of the corpus due to incongruent themes. Nevertheless, the approach reveals the most salient part of the corpus with distinct themes. After identifying topics, the authors manually inspected the topics based on example tweets and created topical labels that describe the major themes in the tweets.

**Table 1:** Tweet count by user clusters.

User cluster	Stage	Number of nonthematic tweets	Total number of tweets included for modeling	Nonthematic tweet ratio
The conservative cluster	1	1041	3094	0.34
The general cluster	1	11,304	31,364	0.36
The progressive cluster	1	1210	4377	0.28
The public health cluster	1	764	1414	0.54
The conservative cluster	2	1565	4711	0.33
The general cluster	2	20,057	43,281	0.46
The progressive cluster	2	3475	10,462	0.33
The public health cluster	2	0	2300	0
The conservative cluster	3	129	430	0.30
The general cluster	3	5309	12,077	0.44
The progressive cluster	3	1120	3539	0.32
The public health cluster	3	0	983	0

### Topics in the Conservative Cluster

The conservative cluster consists of users whose Twitter bios include keywords such as *maga,* *kag*, *trump2020*, *trump*, *conserve*, *patriot,* *wwg1wga*, *2a*, *god*, *Christian*, *nra*, *prolif*, *qanon*, *1a*, *american, constitute, veteran, jesus, proud, country, presid, buildthewal, America, parler, militari, famili, kag2020, vet, draintheswamp, marri, deplor, q, americafirst, usa, backtheblu, wife, freedom, back, truth, retir, ifb, trumptrain, walkaway, dms*, etc*.* These words indicate their alliance with Donald Trump’s campaigns, conservative causes, and religious identities. The cluster also seems US-centric, given that the most central keywords from Twitter bios are associated with US politics. Also notable is the cluster’s tie with the fringe and cult-like QAnon movement. The cluster produced 8235 tweets in stage 1, with approximately 33% of the tweets classified as nonthematic and not included in the following results. Among the tweets included and assigned topics, there are 3600 unique users. Many users (as identified by unique Twitter user IDs) no longer had an accessible Twitter bio in August 2022 (2408 out of 3600), suggesting either that they deleted their accounts or that Twitter suspended their accounts for suspicious activities. Note that tweets sent by suspended accounts and bots, albeit inauthentic, need to be included in the analysis because of their potential polarizing effects. Among those with valid Twitter bios, their follower counts range from 476,284 to 0, with a median of 3860.

**Figure 2:**
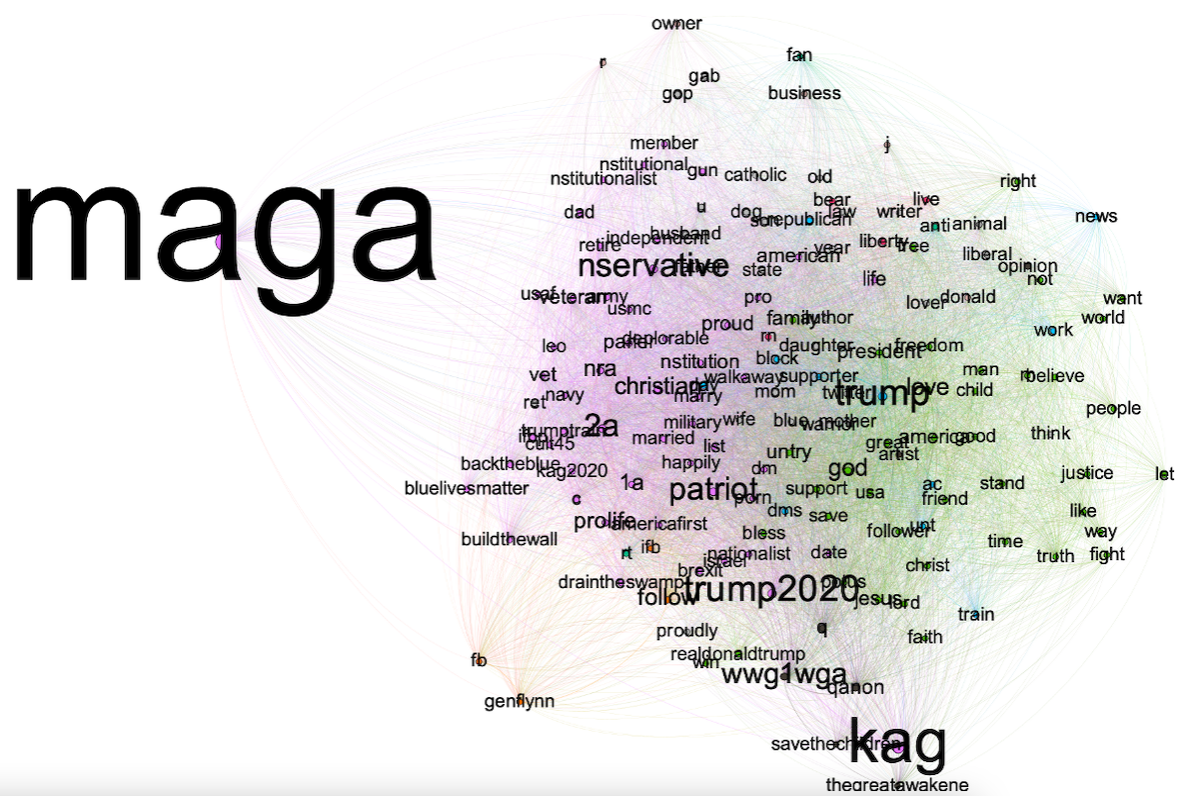
Central terms in Twitter bios of the conservative cluster.

The short text–classification algorithm, *textnets* algorithm, scans all key terms that appear at least twice on the conservative users’ bios and creates a cooccurrence-based semantic network, as seen in Figure 2. Figure 2 shows the top 154 terms ranked by betweenness centrality, a network indicator of key terms’ salience in the entire corpus. Colors in the network graphs indicate distinct thematic clusters. The network shows the central role of Trump-related terms, the purple-colored populist political movements (eg, #BacktheBlue, #AmericanFirst, #DrainTheSwamp, or #WWG1WGA), and the green-colored conservative evangelical community. Table 2 shows the 30 most mentioned locations in the Twitter profiles of this cluster. Note that the data contain user entries on the location fields of their Twitter profile pages. The location information is raw and unstandardized. Specifically, some users may enter detailed cities and states, whereas others may provide general terms such as *United States* or *Planet*. Some could even provide fake or user-created terms to convey one’s politics and ideologies. Such terms include *Real America* or *Hell.* Therefore, the summary statistics about user location entries should be interpreted with caution. Nevertheless, the top entries in the field suggest that users are primarily based in the United States, notably in the most populous US states.

**Table 2:** Top user-entered location information in the conservative cluster.

Location	Value of location
United States	406
Florida, United States	67
California, United States	67
Texas, United States	66
Georgia, United States	30
Virginia, United States	25
Michigan, United States	21
Texas	19
North Carolina, United States	19
Florida	19
Arizona, United States	19
New York, United States	17
Pennsylvania, United States	17
California,	16
Las Vegas, Nevada	15
San Diego, California	14
Missouri, United States	14
Tennessee, United States	13
South Carolina, United States	13
Kentucky, United States	12
Colorado, United States	12
Ohio, United States	11
Pacific Northwest	11
Louisiana, United States	11
Alabama, United States	11
Colorado,	11
Washington DC	10
Phoenix, Arizona	10

**Figure 3:**
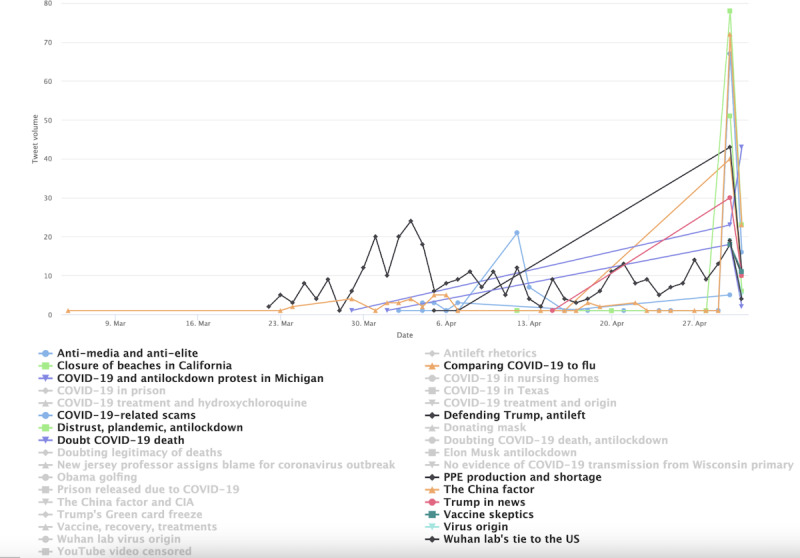
Top topics in the conservative cluster during stage 1.

Figure 3 shows the 10 most salient (denoted by colors) topics in the conservative cluster in stage 1. The less-salient topics were still included in the visualization but grayed out. A small spike was found in early April concerning PPE shortage. The topic’s popularity was overtaken in late April by a broad array of topics suggesting users’ distrust of institutions and resistance to lockdown measures. This includes the third most prominent topic, labeled as “Distrust, plandemic, anti-lockdown,” which peaked in late April 2020 (green colored in Figure 3). One redacted tweet in the topic reads “the who are globalists and are just playing their game f them do not trust they,” which seems to capture the ethos of many similar tweets that display anger toward politicians and the elite. The antiestablishment sentiment is echoed by the fourth most prominent topic labeled “Anti-media and antielite,” which peaked around the same time. The distrust of mainstream media such as CNN is exemplified by this redacted tweet “cnns lemon not holding coronavirus briefings part of the plan for you to think that this is over.” In a similar vein, the fifth-most prominent topic (ie, Doubting COVID-19 death and antilockdown) shows doubts about official statistics on COVID-19 death, as reflected in this tweet example: “...more cancer patients will now die in england because the covid lockdown scared the patients from going to...” and “this is why cause of death is listed as covid even if someone dies of a heart attack it skews the numbers so noon.” The timing of the aforementioned prominent topics also corresponds to the widely reported armed antilockdown protests in the US state of Michigan throughout mid and late April. This specific event is captured by the topic labeled “COVID and anti-lockdown protest in Michigan.” In addition, much of the focus in late April was centered around the Chinese state’s cover-up of the virus in the early days of the pandemic. One such tweet reads, “China knew of virus ability to spread but kept silent for days leaked documents...” Overall, topics prevalent at this stage align with the widely reported Conservatives’ defiance of mask policies and their strong criticism of China in handling the pandemic. The less-prominent topics (the grayed-out topic labels), albeit comparatively small in tweet size, nevertheless shows a diverse range of concerns and interest among the conservative users, such as the alleged laboratory origin of the virus, alternative treatments such as hydroxychloroquine, and skepticism over vaccines.

**Figure 4:**
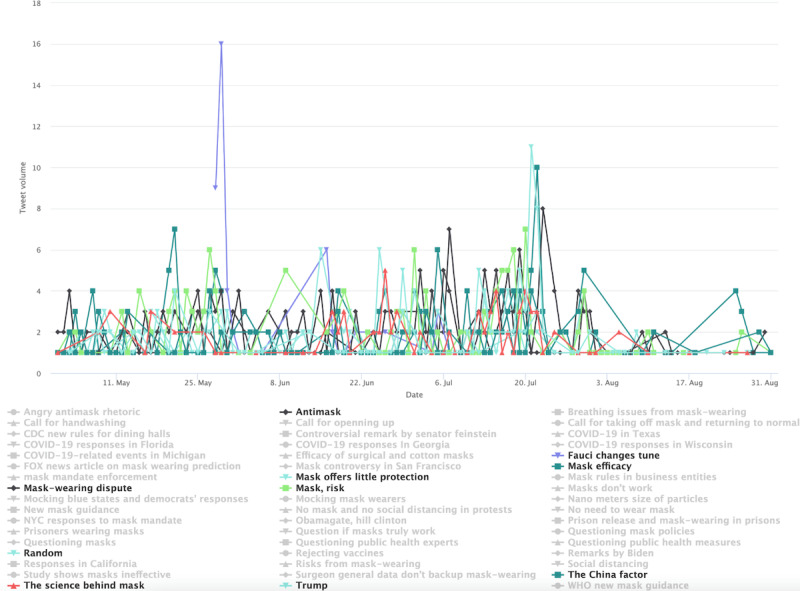
Top topics in the conservative cluster during stage 2.

The conservative cluster’s topics are distinct in stage 2 (Figure 4). Notably, there are common topical clusters about risks associated with mask wearing and the effectiveness of mask wearing in preventing COVID-19. This topical cluster includes topics such as “Mask, risk, mask efficacy” and “Mask offers little protection” as well as the topic labeled “The science behind mask.” Example tweets for these topics include “these face masks will not provide any protection against covid or other viruses or contaminants” and “a cloth mask is as effective fighting covid as a tube sock is preventing pregnancy.” Disputes over masking are also seen in other prominent topics, such as “Mask-wearing dispute,” which contains users’ complaints of having to wear masks for grocery shopping and attending medical appointments. The topic labeled “Mask rules in business entities” includes tweets such as “sheeple are wearing masks like obedient sheep and now stores like walmart require a mask i feel like i am in Orwellian...” which is a clear indication of the users’ resistance to mask wearing. Aside from the 10 most prominent topics, some grayed-out topics (the less-prominent ones by tweet volumes) show spikes and appear politically related. One such spike occurred on May 29, 2020, in relation to Dr Fauci, the lead member of the White House Coronavirus Task Force in 2020, and his stand on masks, which conservative users viewed as inconsistent. One tweet assigned to the topic reads “coronavirus minneapolis faucifraud watch fauci tell you not to wear a mask flashbackfriday” and another that reads “so old fauci was right wearing a mask is useless coronavirus can still pass between face mask wearers even.”

**Figure 5:**
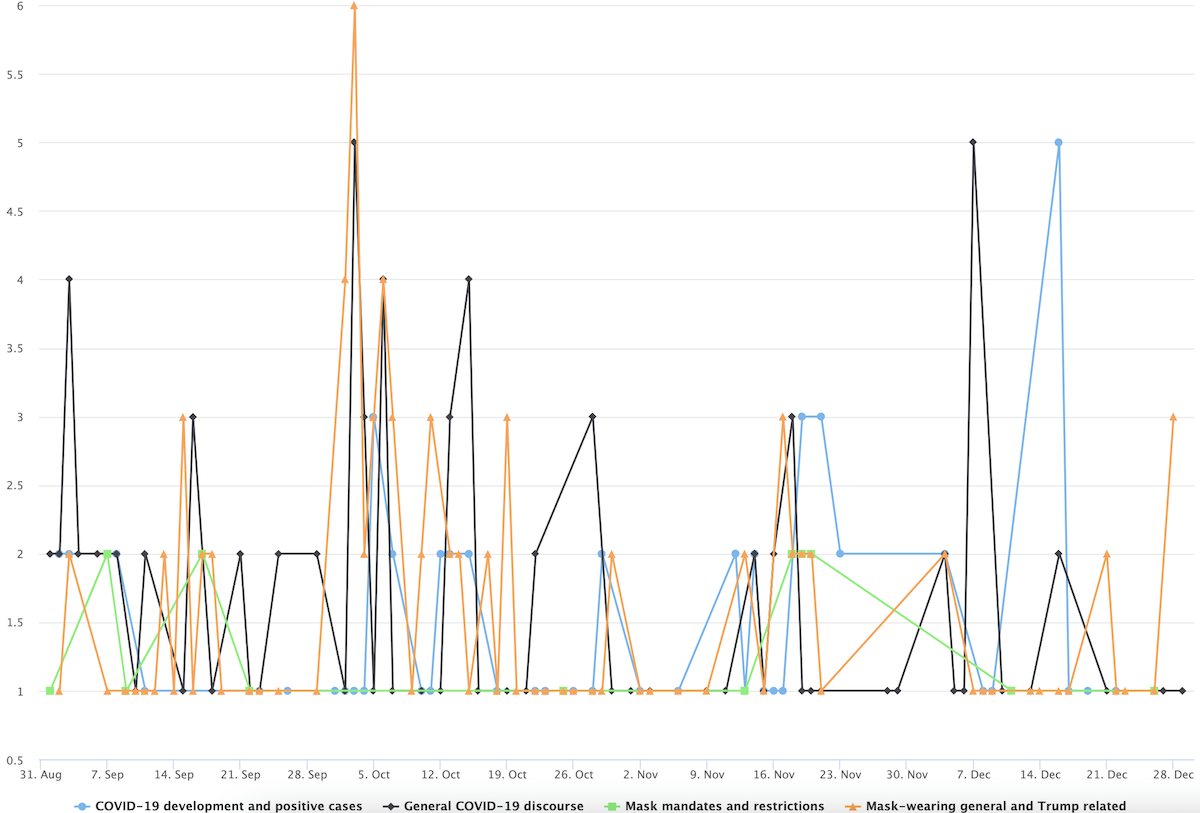
Top topics in the conservative cluster during stage 3.

By stage 3 (Figure 5), the interest in masks by the conservative cluster seems to have dwindled (judging by the sheer tweet volume). Early September was marked by the Conservatives’ focus on Trump and mask wearing, whereas by the later months, the general COVID-19 discourse became more prevalent.

### Topics in the Progressive Cluster

The progressive cluster is distinguishable by marker words on bios such as *resist, fbr, blm, dms, trump, voteblu, theresist, democrat, list, vote, love, anim, lover, biden2020, proud, block, follow, liber, votebluenomatterwho, bidenharris2020, mom, bluewav, retir, blue, dog, pleas, equal, bluewave2020, dm, polit, junki, resist, fbr, news, media, human, social, tweet,* etc. Similar to the conservative cluster, the progressive cluster is centered around US politics and the 2020 election in particular. The cluster produced 18,378 tweets, with approximately 32% of tweets classified as nonthematic and excluded from the final analysis. Among the included tweets, there are 6991 unique users; 1499 of them had invalid Twitter bios in August 2022. The users’ follower size ranges from 5,503,681 to 0, with a median of 4410.

**Figure 6:**
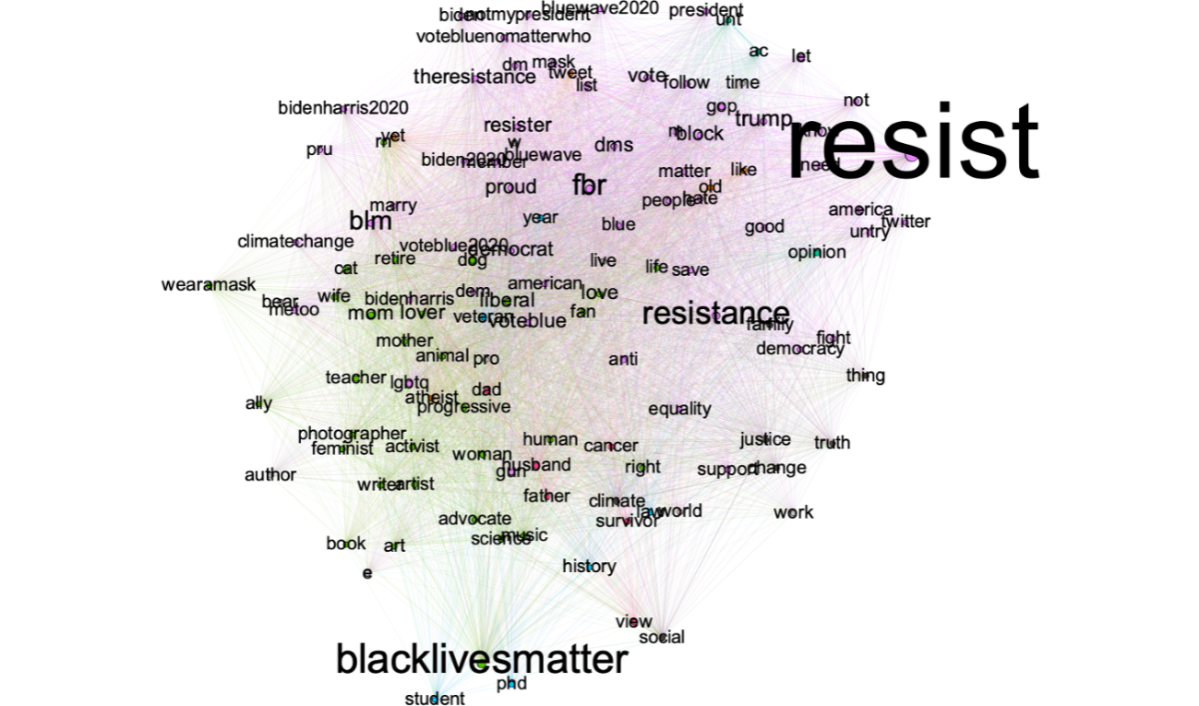
Central terms in Twitter bios of the progressive cluster.

The textnets algorithm (Figure 6) shows 2 distinct clusters: one is tied to progressive social movements such as Black Lives Matter and the Biden Campaign and the second cluster indicates opposition to Trump. Data from the location field (Table 3) indicate US users primarily, particularly in major US metropolitans.

**Figure 7:**
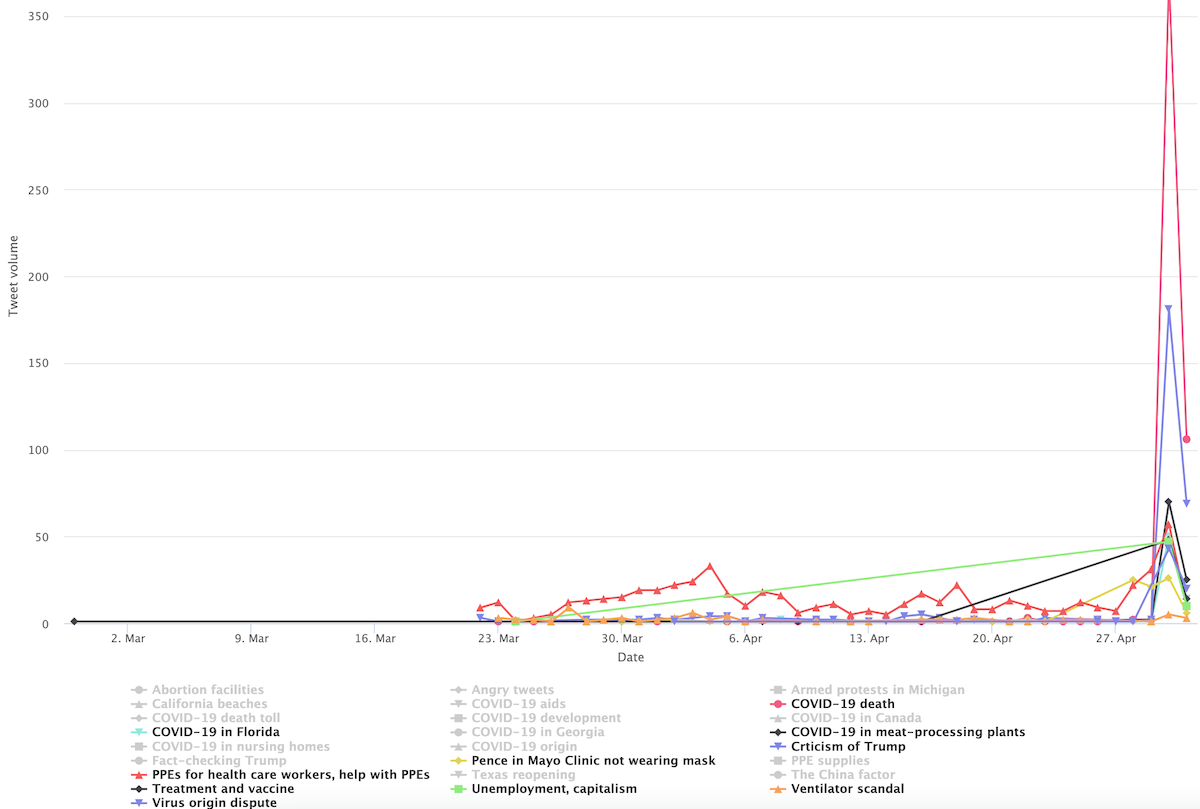
Top topics in the progressive cluster during stage 1.

Figure 7 shows the progressive cluster’s topics in stage 1, such as the conservative cluster, the most prominent topic at this stage concerns the shortage of PPE. The conversation about this topic picked up in early April and it peaked in late April. Example tweets include “the government s emergency stockpile of respirator masks gloves and other medical supplies is running low and...” and “...with having to reuse now either the n masks or the gowns or even the gloves that they are asking us to reuse...” The second-most prominent topic is concerned with COVID-19–related deaths, which registered the biggest peak in the graph on April 30. Some example tweets include “remember when the president fell asleep for a whole month and then poof k dead...” and “all medical workers took an oath and are dying because they believe their oath djt took an oath all of congress...” The third- and fifth-most prominent topics contain criticism of then President Trump and Vice President Pence. One example tweet reads, “trump is losing his mind over reports he is losing his mind this is do or die for trump expect everything...” Another tweet reads, “pence flouts mayo clinic policy by touring coronavirus testing facility without a mask pence defended his actions...” Other prominent topics include outbreaks in different states, and distinct locales (eg, meat processing plants and nursing homes), treatments and vaccines, etc. Similar to the conservative cluster, the China factor and the virus origin were brought up but not to the level of prominence of the Conservatives.

**Table 3:** Top user-entered location information in the progressive cluster.

Location	Value of location
United States	431
California, United States	123
Los Angeles, California	84
Florida, United States	77
Texas, United States	59
New York	49
Texas	42
New York, United States	39
Florida	39
Chicago, Illinois	39
Pennsylvania, United States	38
Earth	38
Ohio, United States	35
New Jersey, United States	34
Washington DC	31
Canada	31
California	30
Dallas, Texas	29
Colorado, United States	27
Virginia, United States	26
San Francisco, California	26
Portland, Oregon	26
North Carolina, United States	26
Oregon, United States	25
Atlanta, Georgia	25
Arizona, United States	25
Seattle, Washington	24
Minnesota, United States	24
Maryland, United States	24

**Figure 8:**
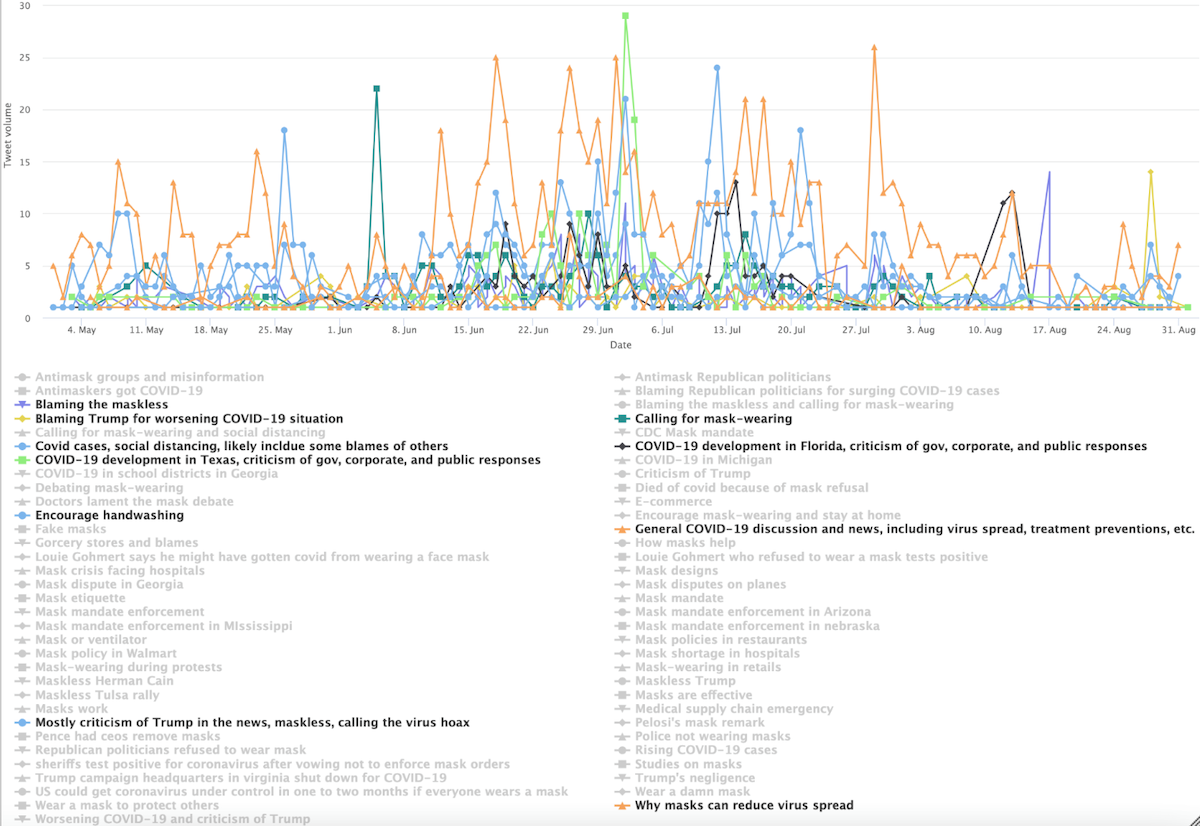
Top topics in the progressive cluster during stage 2.

Entering stage 2 (Figure 8), although general conversations and news sharing about COVID-19 spread dominated the tweets, a notable share of tweets criticized Trump for his negligence in COVID-19 responses and the maskless population for spreading the virus. Example tweets include, “to all the people who think the coronavirus is a hoax humor the scientists and the woke people and wear your god damn mask.” Another reads, “coronavirus cases and covid deaths spike following trump s maskless rally in phoenix more doctors need.” The volume of criticism tweets ebbs and flows, likely reflecting events on the ground. The cluster also includes tweets calling for mask wearing and advocating for the effectiveness of masks. One example reads, “coronavirus cases in florida today please stay safe floridians everybody please wear a mask.” The progressive users’ topical interest also seems to reflect evolving COVID-19 spread across the US states. For instance, a spike was registered on July 2, 2020, following rising cases in Texas.

**Figure 9:**
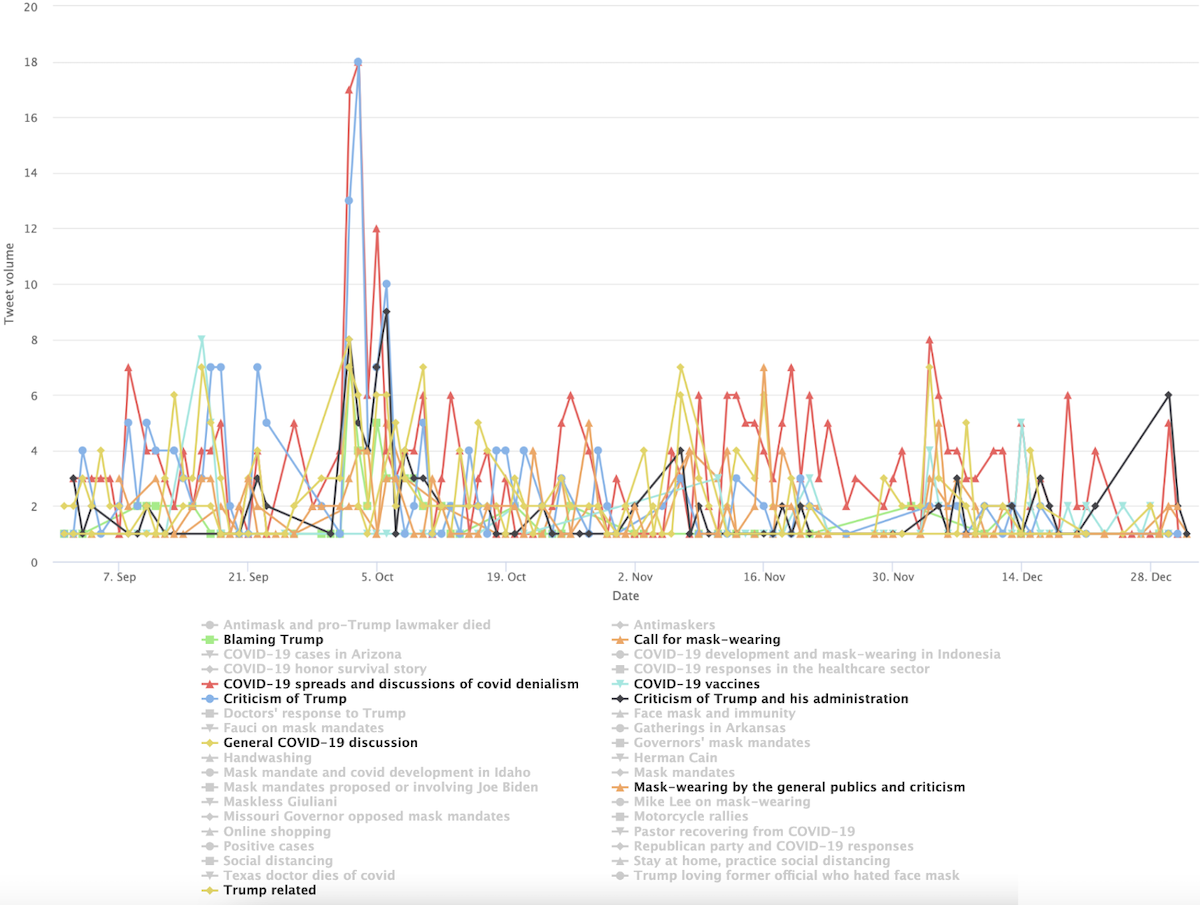
Top topics in the progressive cluster during stage 3.

In stage 3 (Figure 9), the progressive cluster’s conversations were consistently dominated by Trump-related topics, critical of his administration. One notable topic is “COVID spreads and discussions of covid denialism,” which peaked around October 2, 2020. This marked the day when then President Trump tested positive for COVID-19. We also identified a few spikes in volume in topics related to calling for mask wearing and discussions of COVID-19 denialism.

### Topics in the General Cluster

The general cluster consists of users whose Twitter bios contain but are not limited to the following keywords: *tweet, love, world, author, view, work, support, follow, people, former, proud, theresist, writer, us, covid19, research, fan, mom, polit, junki, resist, fbr, news, media, human, social, tweet, right, report, music, opinion*. As the keywords suggest, these users, although they could be interested in politics, do not feature strong partisanship through Twitter profiles. This cluster produced 86,722 tweets, with about 42.3% classified as nonthematic in the topic modeling. There are 33,364 unique users, among which 6973 users did not have a valid and accessible Twitter bio in August 2022. Notably, some accounts affiliated with news media and international organizations were classified into this category (notably World Economic Forum, UNICEF, MSNBC, the partisan influencer Ben Shapiro, and China’s state media CGTN), despite our efforts to exclude a distinct media-affiliated cluster. This means that the general cluster includes both average citizens and some affiliated media. The follower count ranges from 13,280,615 to 0, with a median of 4410.

**Figure 10:**
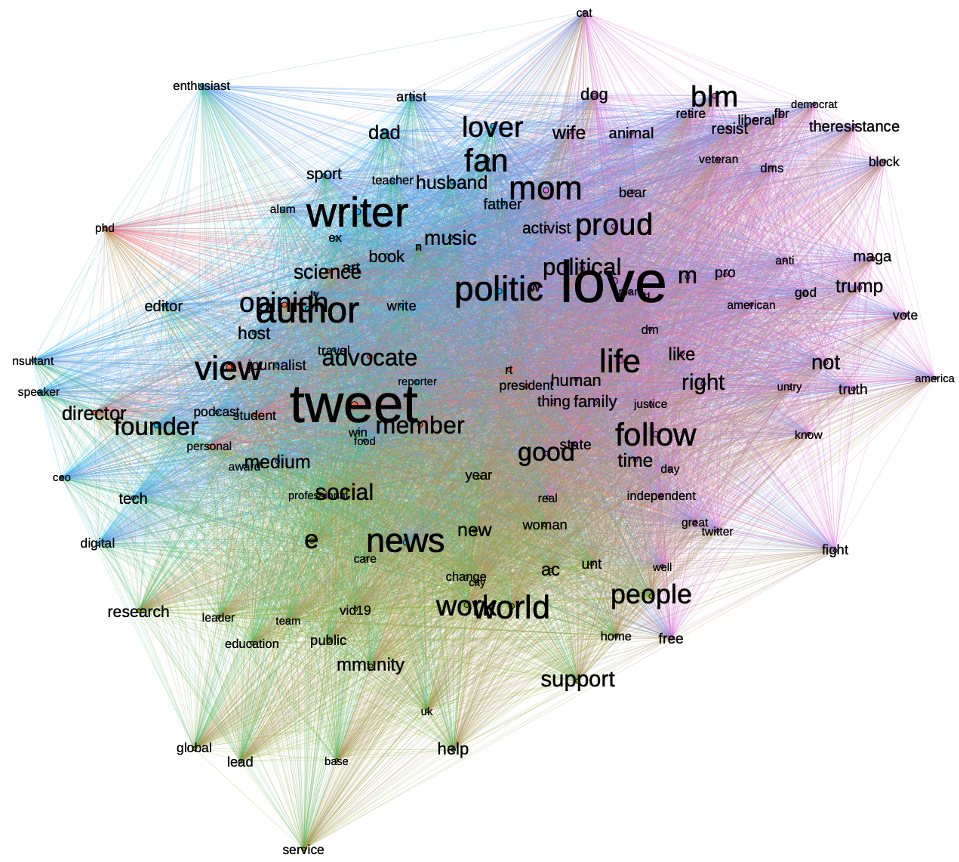
Central terms in Twitter bios of the general user cluster.

Figure 10 shows the top 134 key terms ranked by betweenness centrality. For the keywords to be included in the textnets clustering, they must appear at least 15 times. Figure 10 shows a cluster (blue) based on social and professional roles, a cluster (green) based on news, and a cluster (purple) that contains keywords related to Trump and his campaign. However, it should be noted that Trump-related keywords are not as central as they appear in the Twitter bios of the conservative cluster. The top entries in the location fields (Table 4) show that users in the cluster are primarily based in the United States, residing in major metropolitan areas.

**Figure 11:**
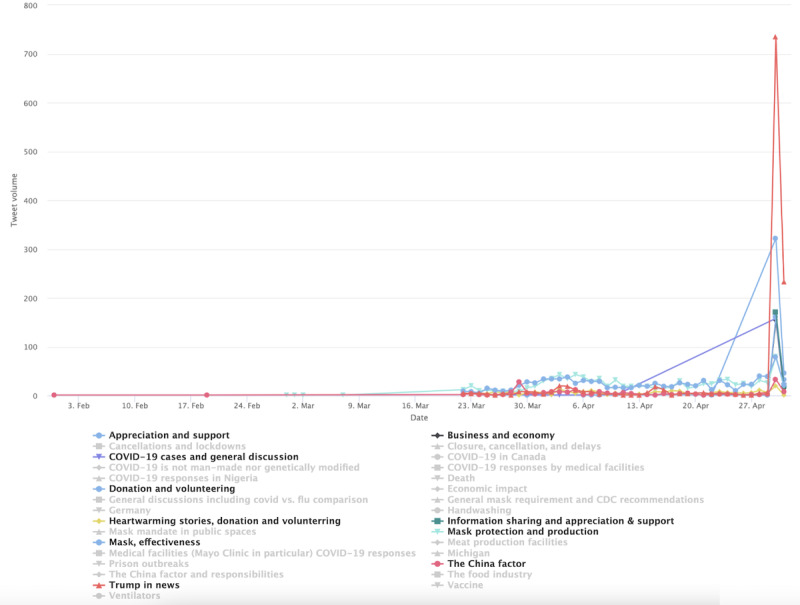
Top topics in the general cluster during stage 1.

Unlike the previous 2 clusters, which are visibly political in Twitter bios and in mask-related tweets, the users in the general cluster indicated various social and professional identities and lifestyles but with peripheral mentions of politics. Therefore, this user cluster is considered less politically inclined than the previous 2 clusters. Their apolitical nature is reflected in mask-related tweets in stage 1 (Figure 11). Although the most prominent topic is about Trump’s responses to COVID-19, other topics do not seem to have a clear partisan slant. Such topics include showing appreciation and support and calling for donations. Example tweets include “we are truly grateful to our heroes in this covid pandemic ayekoo staysafe flattenthecurve” and “thank you to everyone that donated to the covid donation drive for navajo nation today you give me hope.” General users also seem to pay attention to economic impacts and the loss of lives. The roles of China were brought up but less saliently than previously mentioned top topics.

**Table 4:** Top user-entered location information in the general cluster.

Location	Value of location
United States	1758
Washington DC	486
California, United States	417
Los Angeles, California	403
New York	322
Texas, United States	292
Florida, United States	279
London, England	267
London	265
Canada	241
Chicago, Illinois	214
United Kingdom	380
New York, United States	186
Global	177
Boston, Massachusetts	168
Atlanta, Georgia	161
Houston, Texas	153
Worldwide	151
Texas	151
Earth	146
New York	144
San Francisco, California	142
India	140
Seattle, Washington	139
California	139
Toronto, Ontario	137
Austin, Texas	135
Nigeria	129

**Figure 12:**
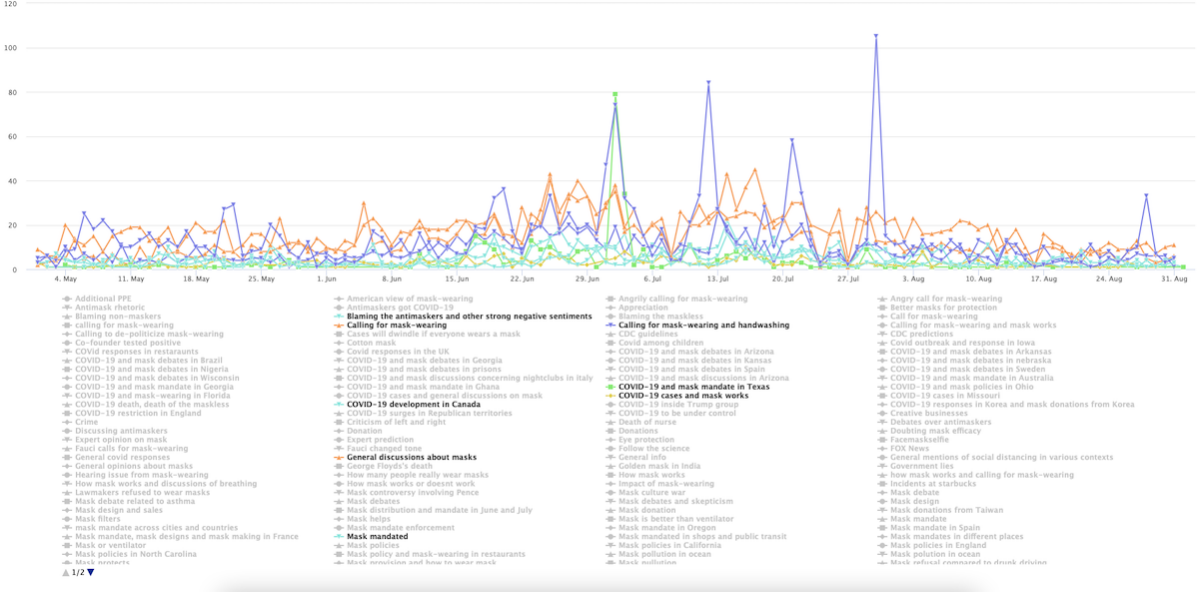
Top topics in the general cluster during stage 2. PPE: personal protective equipment.

Entering stage 2 (Figure 12), the general users’ topics became much more diverse. Although the most prominent topic, labeled “General discussions about masks,” does not seem partisan leaning, the second-most prominent topic is related to news coverage of Trump. The spike on June 20, 2020, corresponds to Trump’s campaign rally in Tulsa, Oklahoma. The spike on July 12 corresponds to the timing when Trump was seen wearing mask in public for the first time. The spike around July 20 corresponds to Trump’s endorsement of masks on Twitter and in media appearances. Other prominent topics include calling for masking and handwashing and blaming antimaskers. However, such topics were overshadowed by the Trump-related topic.

**Figure 13:**
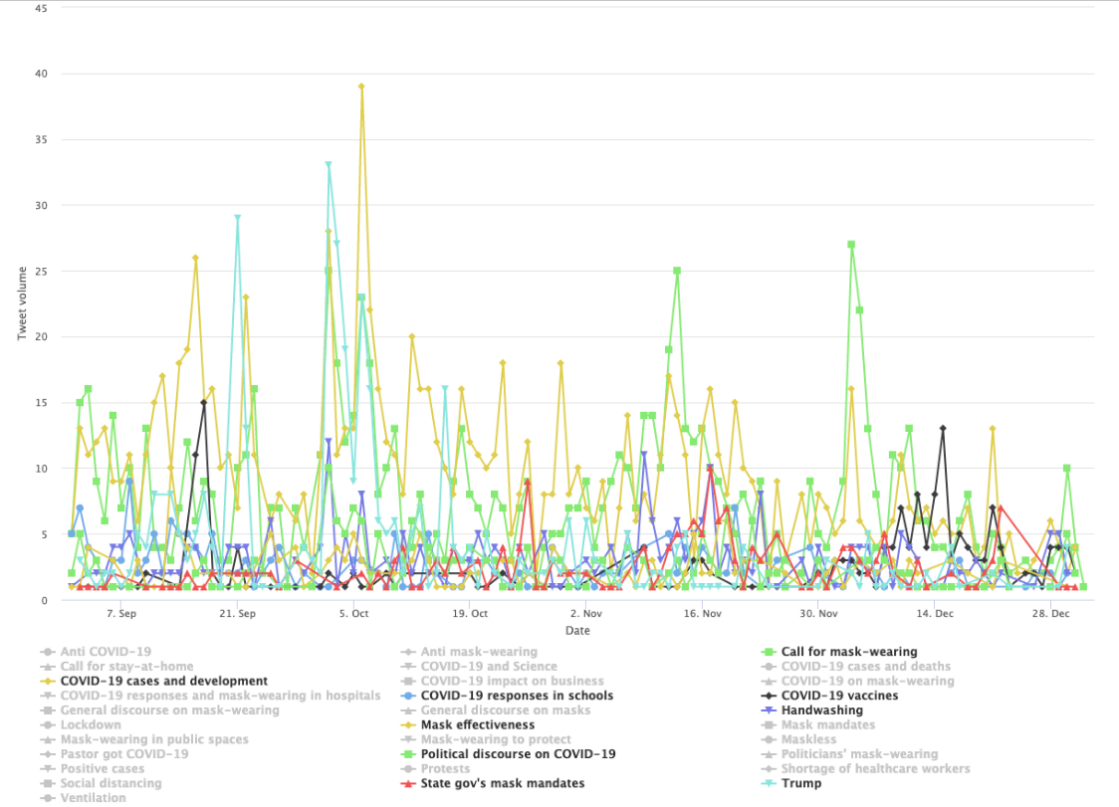
Top topics in the general cluster during stage 3.

In stage 3 (Figure 13), the cluster’s conversation was more general, following several topics identified in previous stages. Such topics include a call for mask wearing and handwashing, general discussion and news sharing about COVID-19 cases, as reflected by the prominence of the topics labeled *COVID cases and development*, which ebbs and flows throughout stage 3. However, Trump-related topics registered several spikes.

### Topics in the Public Health Cluster

**Figure 14:**
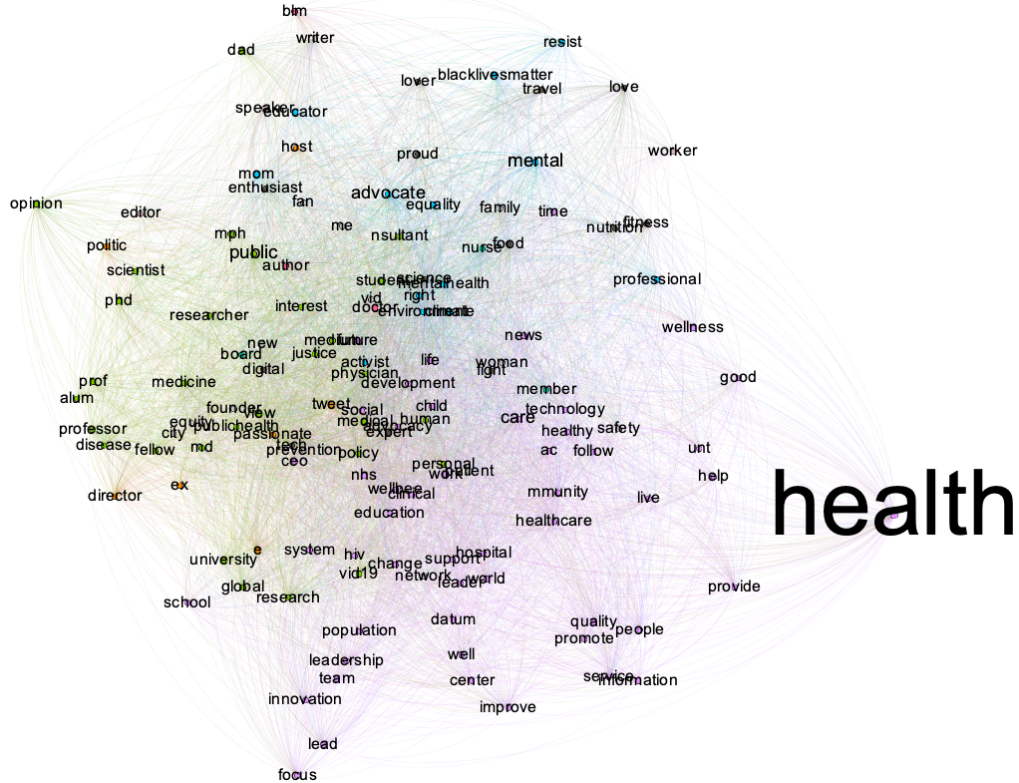
Central terms in Twitter bios of the public health cluster.

Users in the public health cluster are defined by the following bio keywords: *health, care, public, advoc, mental, covid19, research, global, scienc, center, community, improv, view, tweet, polici*. This cluster produced a total of 4697 tweets, with 16.2% classified as nonthematic. The unique user count is 2165, with 13% of them having no valid Twitter bios in August 2022. The follower size ranges from 11,703,587 to 3, with a median of 4413. The textnets algorithm shows clusters by health care specialties and fields, and users seem to be predominantly related to the health care sector. Figure 14 shows the top 137 key terms (which appeared at least twice in the users’ bios) by betweenness centrality. Top entries on the location fields (Table 5) show a more geographically diverse of users compared with other clusters.

**Figure 15:**
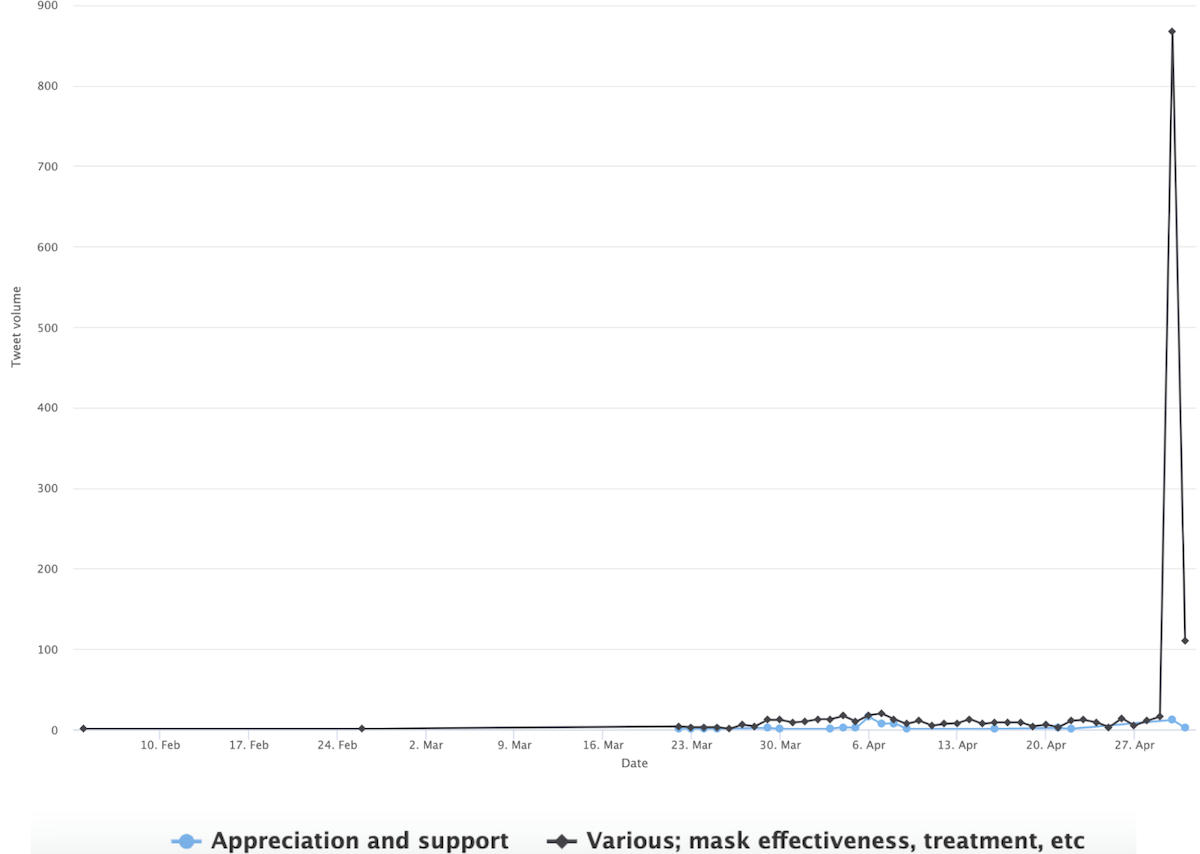
Top topics in the public health cluster during stage 1.

**Figure 16:**
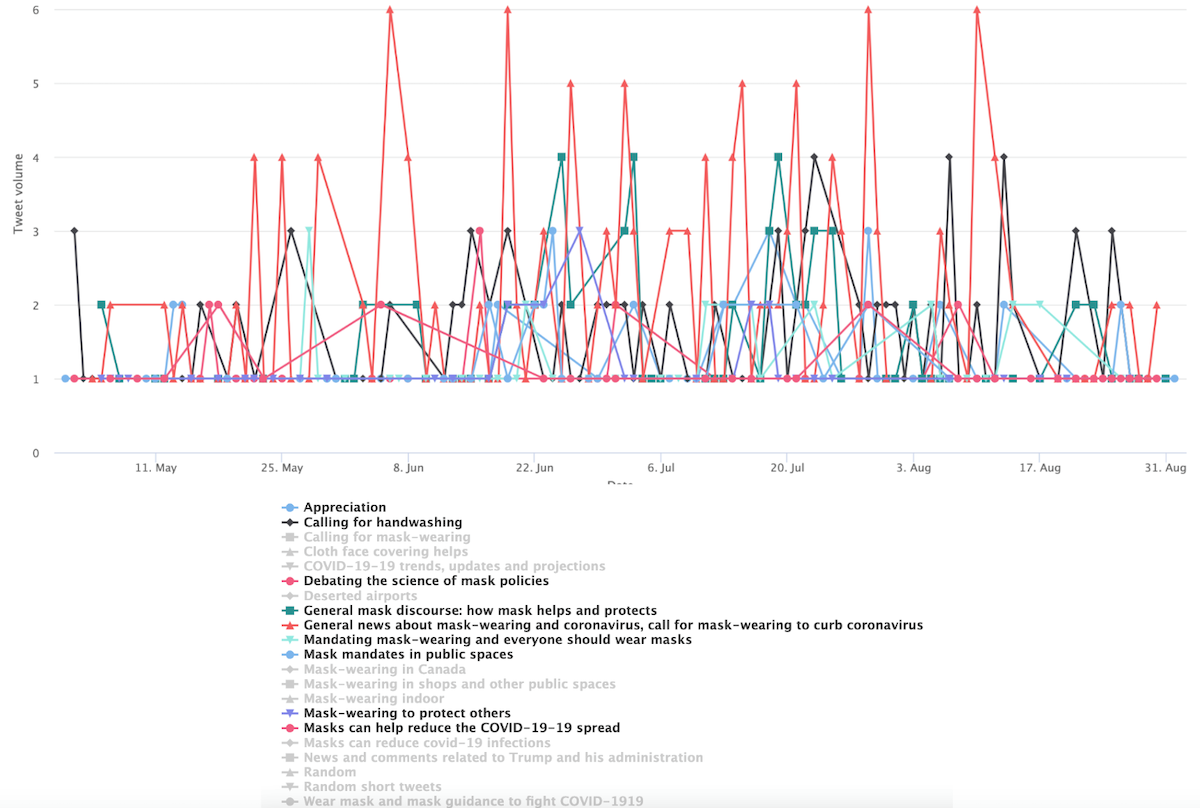
Top topics in the public health cluster during stage 2.

**Figure 17:**
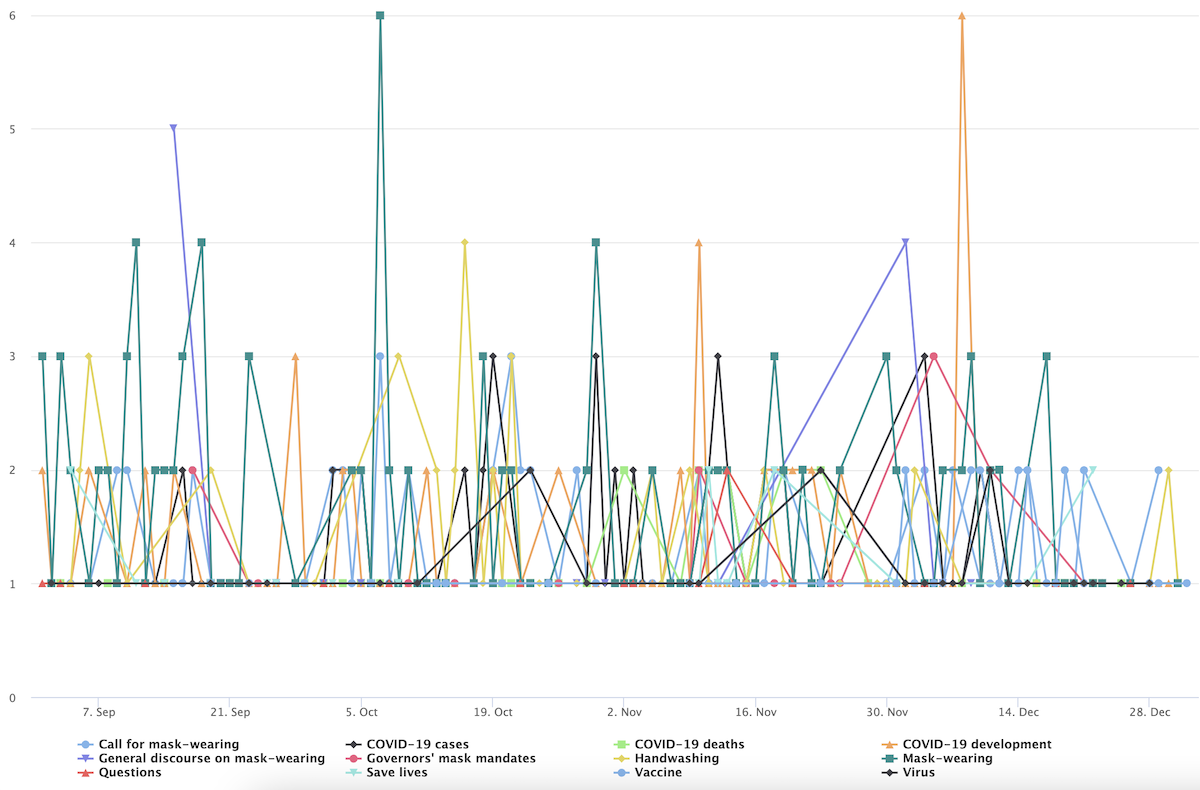
Top topics in the public health cluster during stage 3.

The public health cluster sent fewer tweets than other user clusters, and the cluster produced fewer topics. Early on, their tweets were about showing appreciation (Figure 15) and discussing mask effectiveness such as this tweet “...asymptomatic covid carriers have led the to reconsider its guidelines for who should wear masks...” In stage 2 (Figure 16), the cluster produced a more diverse set of topics, with general news sharing about masks being the most prominent, followed by a cluster of topics that call for handwashing and mask wearing. A similar set of topics were found for stage 3 (Figure 17), centered around a call for mask wearing and handwashing and general discussions about the COVID-19 pandemic development.

**Table 5:** Top user-entered location information in the public health cluster.

Location	Value of location
Washington DC	45
United States	39
Los Angeles, California	23
London, England	21
Canada	19
Global	19
Chicago, Illinois	19
Toronto, Ontario	16
Nigeria	16
London	16
Ann Arbor, Michigan	16
United Kingdom	15
Geneva, Switzerland	13
Boston, Massachusetts	12
California	12
Worldwide	11
United Kingdom	11
Philadelphia, Pennsylvania	11
Seattle, Washington	10
San Diego, California	10
New York	10
Houston, Texas	10
London, United Kingdom	10
Columbus, Ohio	10
Austin, Texas	10
California, United States	10
Toronto, Canada	9
Washington DC	9
New York, United States	9

## Discussion

### Principal Findings

First, our findings echo the importance of a priori user classification in analyzing web-based discourse. As illustrated in the work by Walter et al [48], user clusters can be detected by the type of content they produce on the web. Nevertheless, different from the prior work, which assigns users to clusters by topics, we consider users’ active expressions of social, political, and professional identities on social media profiles as the base for clustering. This ensures that we can compare how users’ discussed topics vary by their expressed identities. As expected, in our data set, users varied by the level of political interest and the spectrum of conservative-to-progressive ideology. In support of prior research identifying issue publics based on distinct political and social identity–related expressions on social media profiles, we found that users in the mask discourse also come from both ends of the political spectrum. Some users were visibly politically motivated, as indicated by mobilizational and identarian hashtags (eg, #kag and #maga). It should be noted that, although politically motivated tweets were plentiful, they remained a minority. By comparison, the general user cluster (those that do not have explicit political expressions on Twitter bio descriptions) constituted the largest cluster. The participation from users in the public health sector was less prominent, implying that much of the public discourse was contributed by either laymen or politically minded individuals rather than public health experts. This finding might point to an expert gap in public health messaging. This finding echoes what is found in previous studies of Twitter discourse concerning alternative treatments of COVID-19. Previous studies show that mainstream medical experts and institutions were less influential than partisan figures [69,70]. Arguably, public health experts’ lesser degree of influence could result from politically motivated public distrust in light of medical populism or the absence of public health voices in this important public sphere. Nevertheless, given the increasingly political nature of mask wearing and mask policies, scientific rather than political voices were much needed in the public sphere. Our findings echo other research that has shown that public policies are politicized in civic discourse [71].

Second, topics did vary by different user clusters. Mask policies have become a sharp point of division between the political left and right in many western democracies. Such divisions in our study mapped onto different topical focuses between the progressive and conservative user clusters. One focused on the criticism of the Trump administration, and the other showed cynicism and skepticism toward public health experts. One attended to the impacts of lockdown, whereas the other tweeted more about COVID-19–induced death. Our topic models broadly reflect the policy preferences and ideological variations in response to mask policies. Equally important to note that political topics also emerged in the general users’ discourse. In particular, the public attention paid to elected officials and their masking practices. This shows how politicians’ behavior could potentially drive or divert public attention to and away from important public health measures. To relate to the concept of medical populism, which has been studied in the context of vaccination and pandemic, our topic model revealed potentially populist discourse that pits people against the elite. This is specifically revealed in the conservative users’ dismissive attitude toward public health experts such as Dr Fauci and the US National Institutes of Health and mainstream media that many view as left-leaning. Although populist-sounding topics did emerge, we caution that they were not the most prominent by tweet volume. To recap, our model was able to pick up critical signals (emergent topics or changes of topics) that should be analyzed further to evaluate public health efforts.

Third, although much of the discourse focused on the impact of COVID-19 and politics behind mask policies, some part of the discourse did appear to focus on the science of mask wearing. All user clusters tweeted about the effectiveness of mask wearing. Identifying these topics is critical because further qualitative analyses can be performed on this specific set of tweets to understand the users’ sources, cited studies, and evidence. Findings could be particularly revealing in tweets from the politically motivated users and the general users because some topics appear to question the effectiveness of mask wearing. Our study showed that, methodologically, our model can pick up signals that may point to important public health discourse that needs to be fact-checked.

Finally, much of the discourse fluctuated with significant political developments that involved then President Trump, the early outbreaks in China, and the controversy surrounding the Wuhan laboratory. For public health monitoring, this again illustrated that public acceptance of public health measures did not occur in a vacuum but interacted with political events on the ground. Our implemented model was able to map out topical evolution over time, thus factoring in how external events influence web-based discourse.

### Limitations

Readers are advised to review the findings with several limitations in mind. First, our sample selection left out tweets that were not retweeted. The nonretweeted may be less influential in message spread but are a significant part of the web-based discourse. In other words, our sample choice may have overlooked a broader discourse on the topic. Second, some tweets may have contained hyperlinked content or embedded images. Public responses to mask-related policies could well be reflected in this embedded content rather than in the plain-tweet text. The clustering based on Twitter bios also left out users who did not explicitly used Twitter bios to express social, professional, and political identities, as well as those whose accounts were deleted or suspended. In addition, a certain percentage of tweets are unclassified (the nonthematic) by BERT. This might be the inherent result of user classification based on Twitter bios. We also caution readers that bots were potentially present in the discourse, although their presence might be minimal. This is because we studied only original tweets (as opposed to retweeted content), and typical bots exclusively retweet others’ content without producing original content. Nevertheless, bot traffic should be distinguished from the genuine citizen-generated Twitter conversations. At the time of the writing, the popular opensource bot-detection tools (eg, *tweetbotornot* and *tweetbotornot2*) were experiencing technical issues due to updates to the Twitter API and the proprietary Botometer presents significant cost barriers for analyzing many Twitter users. We alternatively calculated the ratios of users whose profiles were either deleted or suspended by Twitter, which could give us a glimpse into the potential bot traffic in the corpus. All the factors mentioned above may limit the representativeness of the study finding. We call for future studies to investigate the embedded content and to study tweets. We also deem it is important to study the discourse by regions and narratives. Future studies should compare authentic discourse on Twitter to inauthentic discourse (propagated by bots and trolls) and to media discourse (produced by news media accounts on Twitter). More importantly, we call for comparative methodological work to evaluate various text-classification schemes when applied to infoveillance. Although this study focuses on BERT Topic modeling, whether BERT models do outperform other novel text-classification schemes (such as textnets and ANTMN) is an unanswered question. In addition, future works can compare multiple user-classification schemes, which include the bio-based classification and the thematic personas classification [48].

### Comparison With Prior Work

This work builds upon the existing infoveillance work that uses web-based behavioral data to track public health measures and messaging. This work has the following novelties. It is one of the few studies that has specifically looked at the web-based discourse on mask and mask wearing. It also improved the existing infoveillance framework by conducting a priori user classification and using the BERT Topic modeling, which is optimized for short texts.

### Conclusions

This study improves upon the current infoveillance frame that relies mostly on LDA topic modeling and sentiment analysis. We argue that researchers must first conduct a proper identity and interest-based user classicization to reveal topics emergent in the web-based discourse. This step is lacking in many prior works. We then point out the weakness of the traditional LDA modeling and resort to the much-improved BERTopic. The BERT Topic modeling is optimized for short texts and can reveal longitudinal changes of topics. This implementation has resulted in a more gradient picture of the social media discourse on the issue of mask wearing.

## Abbreviations

ANTMN: Analysis of Topic Model Networks
API: application programming interface
LDA: latent Dirichlet allocation
PPE: personal protective equipment
